# Comparing modeling methods of genomic prediction for growth traits of a tropical timber species, *Shorea macrophylla*

**DOI:** 10.3389/fpls.2023.1241908

**Published:** 2023-10-31

**Authors:** Haruto Akutsu, Mohammad Na’iem, Widiyatno, Sapto Indrioko, Sawitri, Susilo Purnomo, Kentaro Uchiyama, Yoshihiko Tsumura, Naoki Tani

**Affiliations:** ^1^ Graduate School of Science and Technology, University of Tsukuba, Tsukuba, Ibaraki, Japan; ^2^ Faculty of Forestry, Gadjah Mada University, Yogyakarta, Indonesia; ^3^ PT. Sari Bumi Kusuma, Pontianak, West Kalimantan, Indonesia; ^4^ Department of Forest Molecular Genetics and Biotechnology, Forestry and Forest Products Research Institute, Tsukuba, Ibaraki, Japan; ^5^ Faculty of Life and Environmental Sciences, University of Tsukuba, Tsukuba, Ibaraki, Japan; ^6^ Forestry Division, Japan International Research Center for Agricultural Sciences, Tsukuba, Ibaraki, Japan

**Keywords:** dipterocarpaceae, timber species, tree breeding, GWAS, genomic prediction, machine learning

## Abstract

**Introduction:**

*Shorea macrophylla* is a commercially important tropical tree species grown for timber and oil. It is amenable to plantation forestry due to its fast initial growth. Genomic selection (GS) has been used in tree breeding studies to shorten long breeding cycles but has not previously been applied to *S. macrophylla*.

**Methods:**

To build genomic prediction models for GS, leaves and growth trait data were collected from a half-sib progeny population of *S. macrophylla* in Sari Bumi Kusuma forest concession, central Kalimantan, Indonesia. 18037 SNP markers were identified in two ddRAD-seq libraries. Genomic prediction models based on these SNPs were then generated for diameter at breast height and total height in the 7th year from planting (D7 and H7).

**Results and discussion:**

These traits were chosen because of their relatively high narrow-sense genomic heritability and because seven years was considered long enough to assess initial growth. Genomic prediction models were built using 6 methods and their derivatives with the full set of identified SNPs and subsets of 48, 96, and 192 SNPs selected based on the results of a genome-wide association study (GWAS). The GBLUP and RKHS methods gave the highest predictive ability for D7 and H7 with the sets of selected SNPs and showed that D7 has an additive genetic architecture while H7 has an epistatic genetic architecture. LightGBM and CNN1D also achieved high predictive abilities for D7 with 48 and 96 selected SNPs, and for H7 with 96 and 192 selected SNPs, showing that gradient boosting decision trees and deep learning can be useful in genomic prediction. Predictive abilities were higher in H7 when smaller number of SNP subsets selected by GWAS *p*-value was used, However, D7 showed the contrary tendency, which might have originated from the difference in genetic architecture between primary and secondary growth of the species. This study suggests that GS with GWAS-based SNP selection can be used in breeding for non-cultivated tree species to improve initial growth and reduce genotyping costs for next-generation seedlings.

## 1 Introduction

*Shorea macrophylla* belongs to the Dipterocarpaceae, the dominant tree family in the tropical rainforests in Southeast Asia (Ghazoul, 2016). It is commercially important in Borneo as a source of timber and fruit that is harvested to produce oil (Randi et al., 2019). It can also be used in plantation forestry because of its rapid initial growth (Lee et al., 1997). Breeding resources such as progeny and provenance trials are rarely available for tropical tree species with the exception of species such as eucalyptus and teak (Grattapaglia & Kirst, 2008; Corriyanti & Muharyani, 2020). Even when breeding resources exist, their scales and the associated facilities are often limited due to a lack of breeding history. Genomic selection (GS; Meuwissen et al., 2001), which has been put forward as a way to shorten tree breeding cycles (Grattapaglia et al., 2018; Lebedev et al., 2020), is therefore an attractive option for expediting the breeding of tropical species. Another advantage of GS in tree breeding is that a training population can be established without mating families or *a priori* information about the family. GS could therefore greatly accelerate improvement in the focal traits of tropical tree species. Further, the genetic resources of tree species with limited breeding history are those of an almost wild population, so their genetic diversity typically exceeds that of a breeding population (Jones et al., 2006). This study investigates the potential for using GS to accelerate tree breeding in species with limited breeding history and little established collections of breeding resources.

GS has a faster cycle time than pedigree-based breeding because it allows individuals to be evaluated early in their lives by calculating their genomic estimated breeding values (GEBVs) using their genomic information. Many studies have compared the performance (i.e., accuracy) of different methods for predicting GEBVs, which is important because the genetic gains from GS depend on the accuracy of the GEBVs (Lebedev et al., 2020). However, these studies have mainly used parametric methods, which can only account for additive genetic effects, in order to capture inheritable genetic effects for parent selection. However, non-additive effects (i.e., dominant and epistatic effects) are also of interest because their inclusion can increase the accuracy of GEBVs and the prediction of genetic responses (Varona et al., 2018). Accordingly, when using simulated data, the predictive ability achieved with non-parametric methods exceeds that for parametric methods when epistatic genetic effects are present (Ober et al., 2011; González-Camacho et al., 2012; Howard et al., 2014). Some GS studies have used non-parametric methods to account for both additive and non-additive genetic effects (Chen et al., 2019; de Almeida Filho et al., 2019; Abdollahi-Arpanahi et al., 2020; Yan et al., 2021). However, there has been no detailed comparison of parametric and non-parametric methods to identify those providing the most accurate GEBVs.

Machine learning using deep learning (DL) methods has recently been remarkably successful in diverse applications involving the processing and analysis of complex datasets including image recognition and natural language processing. Many traits important in forestry, such as growth, are considered to be complex traits because they are regulated by groups of many polygenes. DL appears to be well-suited to the analysis of such complex traits and is therefore increasingly being used for this purpose (Bellot et al., 2018; Ma et al., 2018; Abdollahi-Arpanahi et al., 2020). Among DL models, convolutional neural networks (CNNs) seem to provide more accurate genomic predictions than multilayer perceptrons (MLPs) (Pérez-Enciso & Zingaretti, 2019), possibly because CNNs apply filters to small parts of the input data and can thus model patterns within each part of the data. This may be advantageous when analyzing genotype data covering most of an entire genome because of the genome’s continuous nature. Additionally, CNNs can accept two-dimensional input data such as images, while other methods can accept only one-dimensional data. This means that information on the nuclear phase can be incorporated into the model. Gradient boosting decision trees (GBDTs) are another class of machine learning methods that perform well in classification, regression, and ranking tasks and are starting to be used in GS studies (Li et al., 2018; Azodi et al., 2019; Abdollahi-Arpanahi et al., 2020). GBDTs rely on gradient boosting, in which learning is achieved through iterative minimization of a loss function using base-learners. GBDTs generate decision trees for the base-learners to make predictions, which construct tree-structed predictive models that split samples using their feature values as criteria. Some GBDT implementations have high computational efficiency and achieve accurate predictions in diverse practical applications.

Genomic prediction models are typically built by genotyping an enormous number of genetic markers covering the whole genome of individuals from a training population, which are then regressed against phenotypic traits. However, to apply such models, it then becomes necessary to genotype thousands of progeny individuals derived from the training population. Because progeny populations are generally much larger than training populations, the cost of such genotyping can be very high. Additionally, this approach suffers from the “large *P*, small *N* problem” because the number of explanatory variables (genetic markers in this case) greatly exceeds the number of observations (individuals in the training population). To overcome these problems, some studies have focused on subsets of genetic markers selected using ranking values. The predictive accuracy of such models can rival or exceed that of conventional models using every available marker (Hiraoka et al., 2018; Li et al., 2018; e Sousa et al., 2019).

This is the second empirical study on GS for Dipterocarpaceae and builds on an earlier work (Sawitri et al., 2020) showing that GS could improve the productivity and quality of *S. platyclados*. To investigate the potential of genomic breeding for *S. macrophylla* and other tropical forestry species with limited breeding resources, we aimed to: (1) identify the methods that yield the highest GEBVs predictability, (2) evaluate the effectiveness of DLs and GBDTs in genomic prediction, and (3) determine the impact of genetic marker selection on predictive accuracy.

## 2 Materials and methods

### 2.1 Collecting plant material and phenotyping

A progeny trial of *S. macrophylla* at the PT. Sari Bumi Kusuma forest concession, Central Kalimantan, Indonesia was targeted for genomic prediction modeling. The progeny trial was established in 2006 using open-pollinated seeds from 94 mother trees in natural populations in the forest concession. The progeny was cultivated in a nursery for ten months and then planted with a 6 x 3 meter spacing. Five trees derived from each mother tree were planted using a Randomized Complete Block Design (RCBD) in a single block and eight block replicates were prepared, giving 3760 progeny trees in total. The phenotypic variance of the progeny population was deemed adequate because significant between-family differences in diameter at breast height (DBH) and total height (HT) were observed 4 years after planting (Widiyatno et al., 2014). Two blocks on relatively gentle terrain containing 940 trees were selected to build genomic prediction models. Three trees from each mother tree were removed for thinning in 2015, 9 years after planting. Leaf samples were collected in 2018 from 361 surviving trees, packed in plastic bags with silica gel, and then transferred to a laboratory at JIRCAS and stored in -30°C freezer until DNA extraction. The HT and DBH of these trees were measured at 1, 2, 3, 4, 7, 9, 11.5, and 12.5 years after planting. HT was measured using a measuring rod up to 11.5 years after planting, then using a Vertex instrument at 12.5 years old. DBH was measured by measurement tape.

### 2.2 DNA sequencing and genotyping

Leaf samples (n=361) were individually pulverized in liquid nitrogen, then total genomic DNA was extracted from 60 mg of the resulting powder using a modified Cetyltrimethylammonium bromide (CTAB) method (Murray & Thompson, 1980). The crude DNA was purified using the NucleoSpin® gDNA Clean-up kit (MACHEREY-NAGEL GmbH & Co. KG, Düren, Germany) following the manufacturer’s instructions. The DNA content of each cleaned-up sample was quantified using a Qubit® dsDNA BR Assay Kit and Qubit® 3.0 Fluorometer (Thermo Fisher Scientific Inc., Waltham, MA, U.S.A.), and the samples were adjusted to a final DNA concentration of 25 ng/mL. Two double digest Restriction-site Associated DNA sequencing (ddRAD-seq) libraries were then generated by digesting 500 ng of the DNA using *Mse*I with *Pst*I or *MluC*I with *Bgl*II (New England Biolabs Inc., Ipswich, MA, U.S.A.), as described by Peterson et al. (2012). *Mse*I and *MluC*I are 4-base cutters, while *Pst*I and *Bgl*II are 6-base cutters. The resulting DNA fragments were then ligated with adapter cassettes at restriction enzyme cleavage sites. Since the 4-base cutters had greater numbers of cleavage sites, the corresponding adapters were designed to form Y-shaped structures that suppressed fragment amplification in cases where *Mse*I or *MluC*I sites were on both sides. Indexed PCR fragments were amplified using KAPA HiFi DNA Polymerase (Kapa Biosystems Inc., Wilmington, MA, U.S.A.) and the Access Array Barcode Library for Illumina® Seqencers―384, Single Direction (Standard BioTools Inc., South San Francisco, CA, U.S.A.) following the manufacturer’s instructions. We then selected 430-470 bp fragments using a 2% agarose gel with the BluePippin system (Sage Science Inc., Beverly, MA, U.S.A.). These size-selected PCR fragments were adjusted to a concentration of 10 nM by assuming an average fragment size of 450 bp. Finally, each library solution was sequenced using 2 lanes on the Illumina Hiseq X platform (illumine, San Diego, CA, USA) to obtain 151 bp paired-end sequences.

dDocent version 2.8.13 (Puritz et al., 2014) was used with default settings for quality filtering, read mapping, and SNP calling on raw reads from Hiseq X sequencing of the *Mse*I/*Pst*I library. Version 2.9.4 of the same software was used to perform the same tasks for the *MluC*I/*Bgl*II library. During the read mapping step, all reads were mapped to the draft genome of *S. leprosula* (Ng et al., 2021) which belongs to the same genus as *S. macrophylla*. The output file in variant call format (VCF), which was named “TotalRawSNPs.vcf”, was then filtered using VCFtools version 0.1.16 (Danecek et al., 2011). SNP filtering was performed using a 10-step procedure. In the first step, samples were filtered by applying the following conditions: missing data ≤ 50%, quality value ≥ 30, minor allele ≥ 3 and depth ≥ 3 (–max-missing 0.5, –minQ 30, –mac 3 and –minDP 3). Second, samples were filtered by the proportion of missing data ≤ 0.65 (F_MISS on out.imiss > 0.65). Third, SNPs were filtered by applying the criteria proportion of non-missing data ≥ 0.95, minor allele frequency ≥ 0.05 and mean depth ≥ 10 (–max-missing 0.95, –maf 0.05 and –min-meanDP 10). Fourth, the vcf file was filtered using dDcent_filters v2.9.4 (Puritz et al., 2018) by applying filters “based on allele balance at heterozygous loci, locus quality, and mapping quality/Depth”; filters “based on overlapping forward and reverse reads”; filters “based on properly paired status” assuming paired-end library; filters “based on high depth and lower than 2*DEPTH quality score”; and filters “based on maximum mean depth” with maximum mean depth cutoff 134. Fifth, we removed SNPs with depths below 60% of the average of groups comprising SNPs within distances of 151 base pairs by applying the filter “based on within locus depth mismatch” from dDocent_filters. This was implemented in a modified part of dDocent_filters because it supports the use of vcf files based on a reference genome. Sixth, we applied a filter based on the significance of deviation from Hardy-Weinberg Equilibrium being ≥ 0.001 (–hwe 0.001). Seventh, the two vcf files for the two libraries were combined using “vcfcombine” in vcflib version 1.0.3 (Garrison et al., 2022) with priority given to the library using *Pst*I and *Bgl*II. Eighth, SNPs were filtered by applying the “Filter Genotype Table Taxa” and “Filter Genotype Table Sites” in TASSEL 5.2 (Bradbury et al., 2007) with the following criteria “Min Proportion of Sites Present” 0.9, “Site Min Count” 60% of number of taxa and “Site Min Allele Freq” 0.05. Ninth, solitary SNPs within each scaffold were removed using R version 4.2.1 (R Core Team, 2020) and the vcfR package version 1.13.0 (Knaus & Grünwald, 2017) for beagle imputation. Then, we used beagle 5.4 (Browning et al., 2018; Browning et al., 2021) with the default effective population size: ne value (100,000) to estimate an appropriate *N*_e_ value. The log2-transformed values of the estimated ne obtained from the log file were divided into classes of 0.1 width, and the most frequent class unless the default value class was considered the appropriate class. Finally, missing data were imputed by beagle 5.4 using a *N*_e_ value of 38,968, which is between the values for the two most frequent classes.

### 2.3 Genetic structure and linkage disequilibrium

We analyzed genetic structure using principal component analysis (PCA). First, SNPs within 1000 bp of one-another were thinned using VCFtools (–thin 1000) to prevent detection of biased structure due to neighboring SNPs. The vcf data were converted to matrices with the following coding: -1 for homozygous reference alleles, 0 for heterozygous alleles, and 1 for homozygous alternative alleles: 1. PCA was performed in R using the “prcomp” function. We also analyzed the linkage disequilibrium of each SNP using TASSEL 5.2 with a window size of 870 to complete all combinations within each scaffold. To obtain a trendline for linkage disequilibrium decay within the inter-SNP distance, the $r^{2}$ values were regressed against the inter-SNP distance using the “loess” function with the following parameter settings: degree = 2, control = loess. control(surface = ”interpolate”). The “span” parameter of the function was optimized using four-fold cross-validation, minimizing the root mean squared error of the regression line and the observed data.

### 2.4 Spatial structure analysis for micro-environmental effects

The observed values of the phenotypic data in the progeny trial were corrected by performing a spatial structure analysis to reduce the impact of micro-environment effects, which could otherwise introduce noise that would reduce the accuracy of the genomic prediction models (Chen et al., 2018). This analysis was implemented in R using the “remlf90” function of the breedR package version 0.12.5 (Munoz & Sanchez, 2020) with a splines model to fit spatial structure along the coordinates of the trees using 12 and 13 knots (default setting). We then subtracted the estimated values due to spatial effects from the observed phenotypic values to obtain the corrected phenotypic values. Outliers among the adjusted phenotypic values were removed using the 5% two-tailed Smirnov-Grubbs test, implemented in R.

### 2.5 Genomic heritability

We analyzed genotype and phenotype data to check heritability and SNP effects on each phenotype. Samples were removed if at least one of them was NA within the same year in these analyses. To identify well-predicted phenotypes, narrow-sense genomic heritability was calculated using the equation: $h^{2}=\sigma_{a}^{2}/\sigma_{y}^{2}$ where $\sigma_{a}^{2}$ is additive genetic variance and $\sigma_{y}^{2}$ is total phenotypic variance, which were averaged over ten iterations for each trait. Variances were obtained from variance components estimated using the Bayesian Ridge Regression (BRR) model, which was implemented in R using “BGLR” function of the BGLR package version 1.1.0 (Pérez & de los Campos, 2014).

### 2.6 Genome-wide association study using all individuals

A genome-wide association study (GWAS) was conducted using DBH and HT in the 7th year (D7 and H7) as the traits of interest. These traits were selected (1) because they have relatively high heritability, (2) to avoid maternal effects expressed strongly after germination and during initial growth, (3) because the observation values of HT in the 11th year were discrete, and (4) since measurements using measurement rods are more accurate than those obtained using the Vertex system.

The aim of the GWAS was to identify SNPs significantly associated with the traits of interest and to verify that both traits are complex, therefore, all individuals were used in the first GWAS. This was done in R using the “mrMLM” function of the mrMLM package version 5.0.1 (Wen et al., 2018) with FASTmrEMMA (Tamba & Zhang, 2018). In this method, a single-locus method is first used to scan the whole genome and calculate *P*-values for each SNP. Second, LOD scores are calculated for strongly trait-associated SNPs; these scores indicate the probability of association between the SNP and quantitative trait loci (QTL) of the trait. Population and kinship structures were flattened using principal components 1 to 4 and a covariance matrix (K) inferred by the genetic markers as default setting of the “mrMLM” function (Wen et al., 2018). *P*-values were corrected by the false discovery rate (FDR) to reduce the incidence of false positive SNPs.

### 2.7 Building genomic prediction models and SNP marker selection

Genomic prediction models were generated using each of 12 methods described in the section 2.8. All models were built using 10 training/validation sets and 10 model construct replicates were generated to assess split effects in a half-sib population. After the split of training/validation sets, we confirmed that all SNP markers maintain their polymorphism in each of training populations (**Table S4**). No model construct replicates were generated for GBLUP because the nature of this method means that all replicates yield identical results. Model building was done by first splitting the full dataset into one training and one validation set with a size ratio of 3:1. Genotype data was coded as specified previously (homozygous reference allele: -1, heterozygous allele: 0, homozygous alternative allele: 1). For CNN2D, nuclear phase data were coded as reference allele: 0 and alternative allele: 1. The phenotype and genotype values in the training data were then normalized using “StandardScaler” in the scikit-learn library. Validation data were also normalized using parameters from training data normalization. These scaling steps were omitted for decision tree methods (RF, LGB, XGB), and for genotype values in GBLUP and CNN2D. Next, each model was built using the training data and validated using the validation data. For hyper-parameter optimization, 3-fold inner cross-validation was performed using “TPESampler” to evaluate sampled parameters, maximizing the Pearson correlation coefficient between observed and predicted values. For comparative purposes, predictive ability was defined as the Pearson correlation coefficient between the observed values and the predicted values for the validation data set.

The second GWAS was performed on the training population to attempt to reduce the number of markers used in the genome prediction model by selecting markers with relatively strong additive genetic effects (**Figure S2**). As the result of the marker selection, the average of percentage sharing SNP markers between the pair of two training populations was 23% for the 48 and 29% for the 192 selected SNP markers for D7 and 25% for the 48 and 28% for the 192 selected SNP markers for H7 (**Tables S5, S6**) The Additional models were generated by selecting the 48, 96, and 192 SNPs with the lowest *P*-values for each training population using FASTmrEMMA. Genomic prediction models were then built for each SNP subset in the same manner as the models for all SNPs. It should be noted that models using all SNPs were not generated using CNN1D and CNN2D because the structures of these methods are unsuitable for models with many SNPs.

### 2.8 Genomic prediction modeling methods used in this study

Genomic best linear unbiased prediction: Genomic best linear unbiased prediction (GBLUP; VanRaden, 2008) was implemented in R using the “mmer” function in the sommer package version 4.1.8 (Covarrubias-Pazaran, 2016; Covarrubias-Pazaran, 2018). An additive relationship matrix was generated using the “A.mat” function in sommer package for use in GBLUP models.

Bayesian linear methods: BayesA and BayesB (Meuwissen et al., 2001), Bayesian LASSO (BL; Park & Casella, 2008), BayesC (Habier et al., 2011), and Bayesian ridge regression (BRR; Pérez & de los Campos, 2014) were implemented in R using the “BGLR” function of the BGLR package. These models all have the form $y=1\mu +\sum_{j=1}^{J}X_{j}\beta_{j}+\epsilon$ where y is the continuous response, μ is the intercept, $X_{j}$ are predictors, $\beta_{j}$ are vectors of effects, and ϵ is residuals. They were mainly characterized by their own prior distribution (Gianola, 2013).

Reproducing kernel Hilbert space regression: Reproducing kernel Hilbert space regression (RKHS; Wahba, 1978) was implemented in R using the “BGLR” function of the BGLR package. The resulting model has the form $y=1\mu +\sum_{l=1}^{L}u_{l}+\epsilon$ where y is the continuous response, μ is the intercept, $u_{l}$ is a kernel matrix of random effects, and ϵ is residuals. The distance matrix for observations was calculated using the “dist” function with the optional method=”euclidean” . The bandwidth parameter was set to 1 when generating the kernel matrix.

Random forest: Random forest (RF; Breiman, 2001) was implemented in Python version 3.9.5 (Van Rossum & Drake, 2009) using “RandomForestRegressor” in scikit-learn library version 1.1.2 (Fabian et al., 2011). RF employs decision trees in which the objective variable is calculated by dividing the samples based on their features. These trees are built repeatedly using different sample sets sampled by bootstrap sampling. Final predictions are then obtained by averaging.

Gradient boosting decision trees: Two GBDT methods, eXtreme Gradient Boosting (XGB; Chen & Guestrin, 2016) and Light Gradient Boosting Machine (LGB; Ke et al., 2017) were implemented in Python using “XGBRegressor” in xgboost library version 1.6.2 and “LGBMRegressor” in lightgbm library version 3.3.2. Because both methods have many (8) parameters to optimize (**Table S1**), hyper-parameter optimization was performed to set their values. “TPESampler” in Optuna library version 3.0.2 (Akiba et al., 2019) was used to identify optimal parameters to avoid overfitting and obtain better predictions. XGB and LGB had several common parameters because both algorithms are decision trees, but each method also had some unique parameters chosen based on their specifications or importance. In particular, “max_depth” and “num_leaves” were considered important and their values were chosen to control tree complexity.

Deep learning: Two deep neural networks, CNN1D and CNN2D, were implemented in Python using the Keras library version 2.8.0 (Chollet, 2015). Their architectures are detailed in **Table S3** and were inspired by the model of Simonyan and Zisserman (2015) that achieved remarkable success in image recognition. CNN2D differed from CNN1D with respect to input dimensions, convolution, and pooling layers because they have two dimensions in their nuclear phase. The nuclear phase dimension was summarized in the final pooling layer. CNN1D and CNN2D also had 7 common parameters that were optimized by Optuna (**Table S2**) using “TPESampler” due to their common architecture.

## 3 Results

### 3.1 Genotyping and genetic analysis

278,898,826,194 bases on 1,847,012,094 reads and 279,470,755,304 bases on 1,850,799,704 reads were obtained from the *Mse*I/*Pst*I and *MluC*I/*Bgl*II libraries, respectively, using the Illumina HiSeq X platform. After performing separate assembly and filtering steps for each library using the dDocent pipeline, the *Mse*I/*Pst*I and *MluC*I/*Bgl*II libraries produced 6,516,667 sites from 352 samples and 10,217,939 sites from 368 samples, respectively, which were stored in vcf file format. In the filtering step, some individuals were removed due to excessive missing data or mislabeling. The final vcf files for the *Mse*I/*Pst*I and *MluC*I/*Bgl*II libraries contained data on 11,425 and 10,605 polymorphic sites from 290 samples and were merged into a single vcf file that was then filtered using TASSEL 5.2 and imputed by beagle 5.4 to generate a vcf file representing 18,037 polymorphic sites from 290 samples that was used in all subsequent analyses.

**Figure 1:**
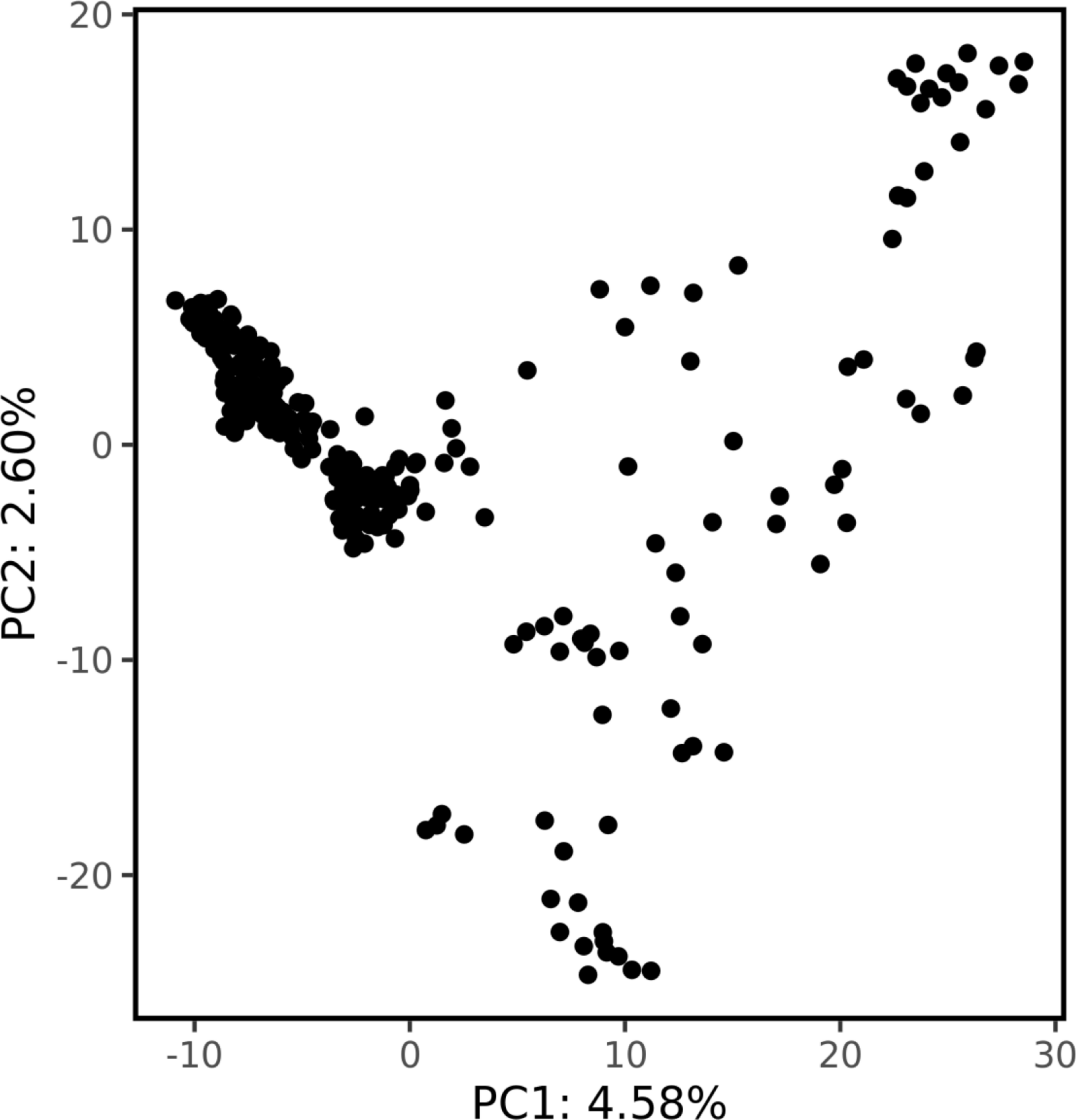
The population structure revealed by principal component analysis. The proportion of the total variance explained by each principal component is shown on the axes.

**Figure 2:**
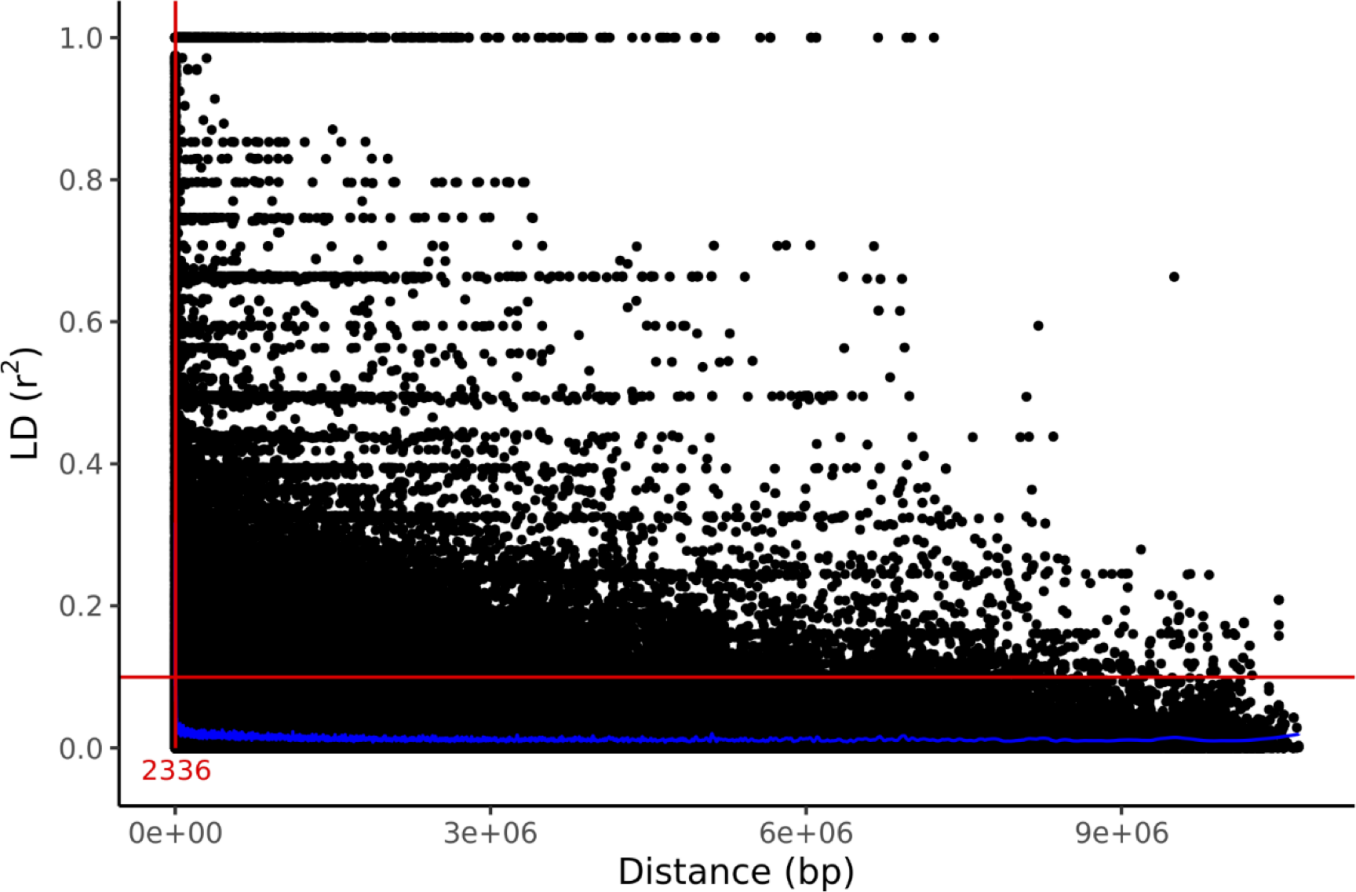
LD decay in the studied population indicated by a scatter plot of $r^{2}$ against the inter-SNP distance. The figure also shows the LOESS regression line (in blue), an intercept at $r^{2}=0.1$ , and the distance at their intersection (red).

Genetic structure was assessed by PCA using 1000 bp thinned genotype data containing 8709 SNPs. The first two principal components (PC1 and PC2) explained 4.58% and 2.60% of the genetic variance, respectively. Additionally, clusters of samples were observed in the left (PC1) and central (PC2) regions of the space (**Figure 1**). Linkage disequilibrium decay was assessed based on pairwise $r^{2}$ values between all SNPs located on the same scaffold. **Figure 2** plots these $r^{2}$ values against the corresponding inter-SNP distances in base pairs and shows the associated trendline. An $r^{2}$ intercept line at 0.1 is also shown to estimate the LD decay of the *S. macrophylla* population. The regression line intersects this intercept line at a distance of 2336 bp.

### 3.2 Spatial analysis of phenotypic traits

**Figure 3:**
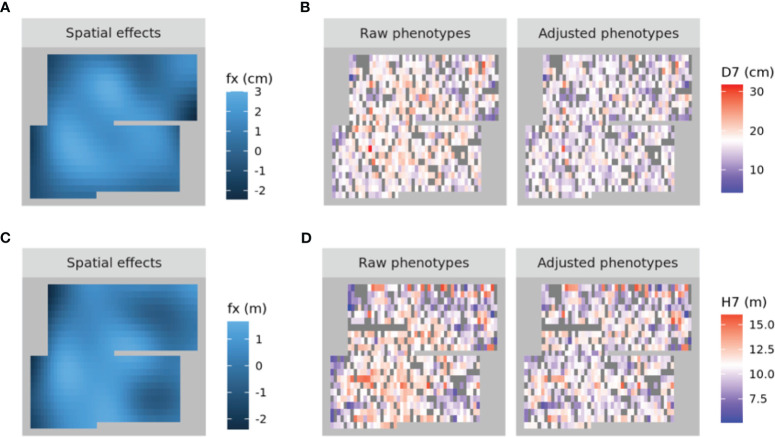
Heatmaps of the spatial effects for D7 **(A)** and H7 **(C)** as well as the corresponding raw phenotypes and adjusted phenotypes (**B, D**, respectively). In **(A)** and **(C)**, higher to lower values are represented by shades ranging from pale to dark blue. In **(B)** and **(D)**, higher to lower values are represented by shades ranging from red to blue, with white denoting intermediate values. NA values are shown in dark grey.

**Figure 4:**
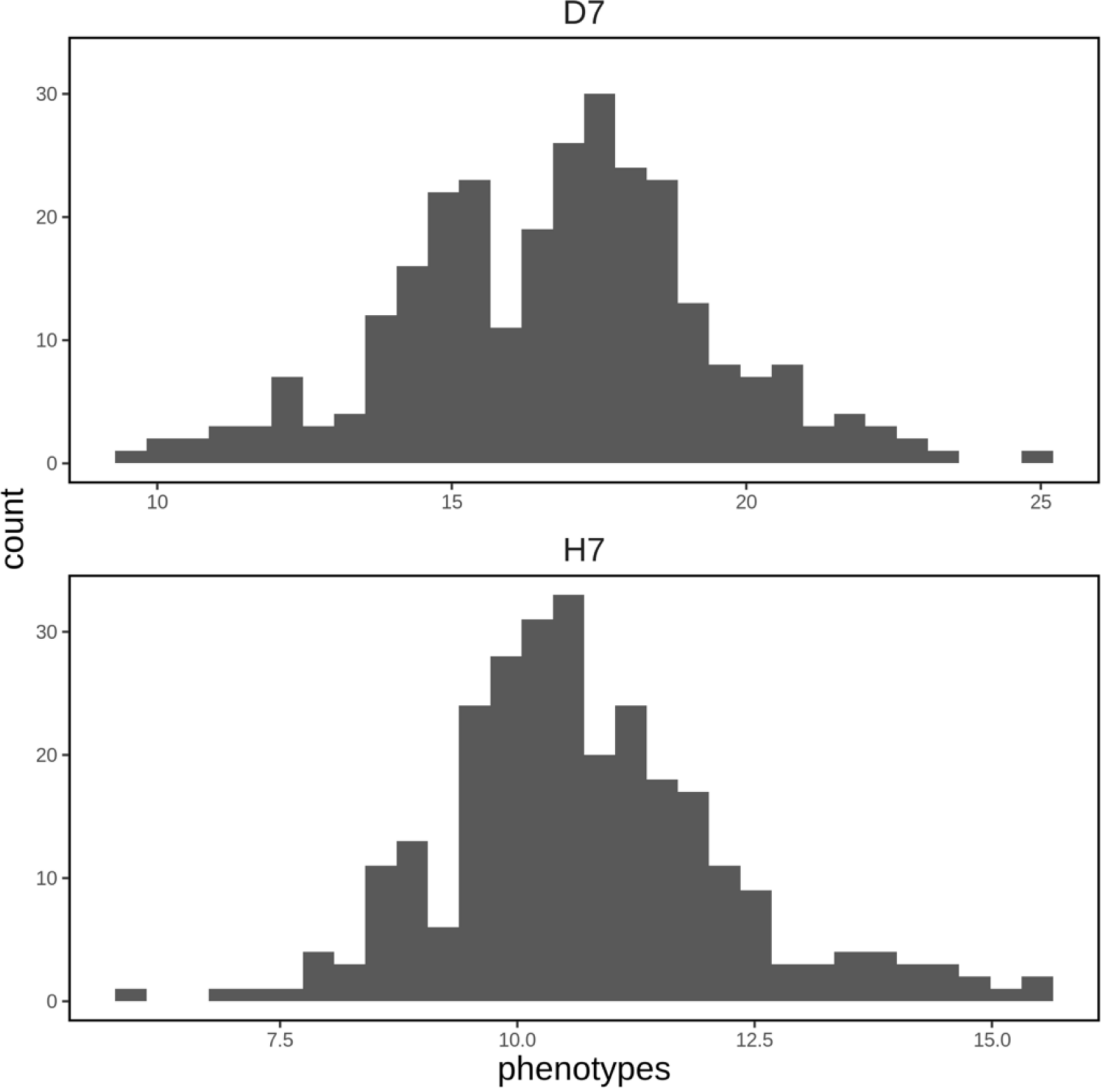
Histograms of adjusted phenotypes for D7 and H7 that were used to construct the genomic prediction model. The units of the horizontal axis are cm for D7 and m for H7.

A spatial structure analysis using a two-dimensional spline model was performed to detect and remove spatial bias in both focal traits (DBH and HT). Trees with lower values of both traits were concentrated on the left side of the progeny trial, which was captured by the analysis and the estimated spatial effect values were also smaller in this area. On the other hand, trees located around the center of the trial had higher phenotypic and spatial effect values. On the right side of the trial, there were some trees whose phenotypic values were lower, accentuating the relatively high phenotypic values of both traits in trees at the center of the trial area. Since the planting locations of progenies from each mother tree were arranged according to a RCBD, the spatial effects observed in the trial can be largely attributed to environmental heterogeneity. We therefore adjusted the raw phenotypic values to minimize the impact of this heterogeneity. The raw phenotypes, spatial effects, and adjusted phenotypes for D7 and H7 were visualized using heatmaps (**Figure 3**). The adjusted seventh-year phenotypes of trees with genotyping data were also visualized using histograms (**Figure 4**).

### 3.3 Genomic heritability

Narrow-sense genomic heritability for DBH and HT from year 1 to year 12.5 was calculated based on variance components obtained from Bayesian ridge regression for sample sets without missing data (**Table 1**). The highest genomic heritability values were 0.406 for DBH in year 1 and 0.395 for HT in year 11.5. The lowest values were 0.288 for DBH and 0.271 for HT, both in year 12.5. The narrow-sense genomic heritability of DBH was always higher than that of HT except in years 2 and 11.5.

**Table 1:** Narrow-sense genomic heritability for diameter at breast height (DBH) and tree height (HT) from year 1 to year 12.5.

year	1	2	3	4	7	9	11.5	12.5
DBH	**0.406**	0.304	0.312	0.373	0.366	0.350	0.391	0.288
HT	0.303	0.324	0.284	0.304	0.358	0.312	**0.395**	0.271

The highest genomic heritability values on DBH and HT were shown in bold.

### 3.4 Genome-wide association study using all individuals

**Figure 5:**
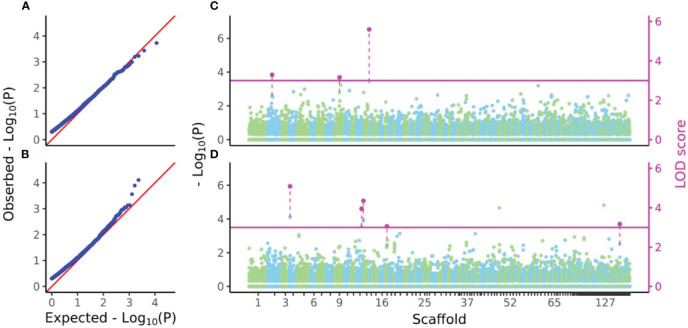
Q-Q and Manhattan plots of D7 and H7. **(A, B)** Q-Q plots of $-{\text{log}}_{10}\left(P\right)$ values from GWAS of D7 and H7. **(C, D)** Manhattan plots of $-{\text{log}}_{10}\left(P\right)$ values and LOD scores from FASTmrEMMA of D7 and H7. In **(A)** and **(B)**, zero values were removed from all $-{\text{log}}_{10}\left(P\right)$ values before calculation of expected values. In **(C)** and **(D)**, all $-{\text{log}}_{10}\left(P\right)$ values of SNPs were separately plotted in light green and sky blue in color which represents localization of SNPs to each scaffold. Only LOD scores above the threshold 3 were shown in magenta and connected by dashed line with the plots of $-{\text{log}}_{10}\left(P\right)$ values of the same SNPs.

*P*-values and LOD scores of SNPs for D7 and H7 were calculated using FASTmrEMMA for the sample sets considered in the genomic heritability analysis. The results were visualized using a Q-Q plot and a Manhattan plot showing all *P*-values and LOD scores above 3, which was taken as the significance threshold (**Figure 5**). For D7, three SNPs had significant LOD scores but non-significant $-{\text{log}}_{10}\left(P\right)$ values (4.0052, 3.8663 and 6.4149) at the 5% statistical significance level after FDR correction. For H7, five SNPs had significant LOD scores but non-significant $-{\text{log}}_{10}\left(P\right)$ values (5.8854, 4.7025, 5.1203, 3.7573, and 3.88) at the 5% statistical significance level after FDR correction (**Figure 5**).

### 3.5 Comparison of genomic prediction modeling methods

8480 genomic prediction models were built using 12 models (with 10 model building replicates, 10 sample split replicates, 4 different numbers of selected SNPs based on the *p* value of the second GWAS using each of the training populations, and 2 focal traits per method). The median of predictive abilities within model building replicates and sample split replicates were positive and majority of the predictive abilities was significantly deviated from zero except CNN1D of D7 (96, 192, all SNPs), GBLUP of H7 (all SNPs), LGB of D7 (all SNPs), RKHS of D7 (96 SNPs), XGB of D7 (all SNPs), XGB of H7 (all SNPs) (**Table 2**, **Table S7**). The highest median predictive ability for D7 was 0.188, which was achieved using RF with the full set of SNPs. For H7, the highest median predictive ability was 0.231, which was achieved using RKHS with 48 selected SNPs. Predictive accuracies could not be calculated for H7 using three of the GBLUP models using the full set of SNPs because their predictions were identical to those for the test set (shown in parentheses in **Table 2**). When the median of predictive accuracy was compared among the selected number of markers, the higher number of SNPs tended to yield higher estimation accuracy for D7 for all methods except LGB and CNN2D. On the other hand, for H7, a lower number of SNPs tended to give higher estimation accuracy for all methods.

Table 2Median predictive ability values of genomic prediction models when used with 48, 96, and 192 or with the full set of 18037 SNPs.

**Table:** 

Traits	Number of SNPs	Predictive ability (median)					
		GBLUP	BayesA	BayesB	BayesC	BRR	BL
D7	48	0.100	0.087	0.086	0.085	0.087	0.088
	96	0.125	0.116	0.122	0.121	0.113	0.119
	192	**0.176**	0.154	0.152	0.156	0.157	0.152
	18037	0.130	0.134	0.133	0.134	0.132	0.136
H7	48	0.206	0.211	0.200	0.200	**0.212**	0.208
	96	0.201	0.209	0.206	0.204	0.207	0.209
	192	0.160	0.157	0.174	0.167	0.146	0.167
	18037	0.056	0.110	0.108	0.109	0.108	0.107

**Table:** 

Traits	Number of SNPs	Accuracy (median)					
		RKHS	RF	LGB	XGB	CNN1D	CNN2D
D7	48	0.086	0.121	**0.167**	0.119	0.062	0.108
	96	0.106	0.125	**0.142**	0.093	0.039	0.073
	192	0.136	0.164	0.155	0.120	0.082	0.098
	18037	0.140	**0.188**	0.119	0.090	–	–
H7	48	**0.231**	0.204	0.162	0.181	0.179	0.184
	96	0.209	0.195	0.157	0.191	**0.218**	0.199
	192	0.159	0.165	0.142	0.148	**0.200**	0.173
	18037	0.125	**0.151**	0.092	0.075	–	–

Abbreviations of methods are as follows: GBLUP for genomic best linear unbiased prediction, BRR for Bayesian ridge regression, BL for Bayesian LASSO, RF for random forest, LGB for light gradient boosting machine, XGB for extreme gradient boosting, CNN1D for convolutional neural network for one-dimensional input and CNN2D for convolutional neural network for two-dimensional input. Predictive ability was defined as the correlation coefficient between the observed and predicted values for each model. The tabulated values are medians of 100 replicates based on 10 pairs of training and validation sets and 10 model construction replicates. The highest accuracy values for each trait and number of SNPs are shown in bold.

## 4 Discussion

### 4.1 Linkage disequilibrium analysis and genome-wide association study

After accounting for linkage disequilibrium decay, the approximate genomic coverage was estimated to be 2236×8709=19,473,324 bp based on the intersection of the regression line and the $r^{2}=0.1$ line (2336 bp) and the number of SNPs (8709 SNPs) after thinning the genotype data at every 1000 bp. The *S. macrophylla* genome size was estimated to be 363.816 Mbp using the formula of Doležel et al. (2003) because the *C*-value of *S. macrophylla* is 0.372 (Ng et al., 2016). Therefore, the genotype data in this study represents around 5.35% of the total genome. The GWAS for D7 and H7 detected 3 and 5 SNPs with LOD scores above 3, respectively, but no significant SNPs were detected in terms of *P*-values (**Figure 5**). The inability to identify QTLs for D7 and H7 may be due to the sparsity of marker coverage within the genome and the fact that both traits are probably regulated by complex genetic backgrounds that are not readily characterized. In order to obtain ideal results in GWAS, it is necessary to further increase the marker density and the power of LD detection by increasing the number of individuals.

### 4.2 Comparison of methods for building genomic prediction models and population subdivision

GBLUP achieved maximum predictive abilities of 0.176 for D7 and 0.206 for H7. Notably, when considering only results with selected SNPs, the value for D7 was the highest achieved with any method in this work (**Table 2**). Similarly, in a GS study on Japanese cedar, GBLUP achieved greater accuracy than BayesB for most traits including growth, wood properties, and male fertility. BayesB is considered to be effective only in cases involving a small number of strongly linked QTLs (Jannink et al., 2010), so the superior performance of GBLUP suggests that the focal traits in this work are governed by large numbers of QTLs (Hiraoka et al., 2018). Therefore, the selected SNPs (192) are possibly linked to many QTLs in D7, which contributes to the higher predictive ability of GBLUP than BayesB. Among the linear Bayesian methods (BayesA, BayesB, BayesC, BRR, and BL), BRR had the best predictive ability for both D7 (0.157) and H7 (0.212) but only outperformed the next best models (BayesC and BayesA, respectively) by 0.001 for both traits, and there were only minor differences in accuracy between all of these methods with the exception of BayesB (**Table 2**). An earlier study on cattle similarly found no difference in predictive ability between the Bayesian methods, which was consistent with a simulated scenario involving multiple QTLs (Foroutaifar, 2020). BayesB achieved a higher predictive ability for H7 when using a relatively large number of SNPs (192 SNPs) and a lower accuracy when using a relatively small number of SNPs (48 SNPs) when compared to other Bayesian linear methods. BayesB has also proved to be an effective method for traits dominated by QTL with large effects in several studies (Daetwyler et al., 2010; Wang et al., 2019). We therefore suggest that the difference between BayesB and other Bayesian linear methods was due to its reliance on the assumption that there were many SNPs with no genetic variance (Meuwissen et al., 2001). RKHS achieved a maximum predictive ability of 0.140 for D7 and 0.231 for H7 and offered the best performance for H7 of all the tested methods (**Table 2**). This is consistent with an earlier GS study on pine, where RKHS was slightly more accurate than BayesA for DBH, HT, and simulated traits including non-additive genetic effects. This outcome is also consistent with the argument that RKHS non-explicitly accounts for non-additive genetic effects (de Almeida Filho et al., 2019) and suggests that such effects have an important influence on H7. The predictive ability achieved for H7 when using RKHS was only slightly sensitive to the number of selected SNPs but was highest when using only 48. This may be because the relative influence of non-additive genetic effects was increased when restricting the analysis to the SNPs with the largest effects. RF achieved a maximum predictive ability of 0.188 for D7 when using the full set of SNPs, and 0.204 for H7 when using 48 selected SNPs. RF achieved the highest predictive ability for D7 out of all the methods tested in this work. The predictive ability for H7 was always highest when using the full set of SNPs, irrespective of the choice of method (**Table 2**). RF also achieved better accuracy than BayesB and GBLUP in genomic predictions for cedar growth and woody strength (Hiraoka et al., 2018). Nonlinear methods such as RF should be more effective when the relationship between the feature and objective variables is nonlinear, which is expected to be the case when epistatic effects account for the majority of genetic variance (Jannink et al., 2010). D7 and H7 may thus have epistatic genetic structures.

No studies have yet investigated whether observed GP model performance can reliably be extrapolated to next generation of a training population. However, since the test population in this work consisted of just 71 individuals, it is very likely that predictive ability was influenced by the relationship between the training and test populations. In an earlier study on maize, the highest predictive ability was achieved with models based on a dataset in which both parents were shared by the training and test populations, followed by a dataset where only the mothers were shared. Performance was significantly worse for datasets that shared only fathers or with no shared parents (Yan et al., 2021). The progeny trial examined in this work was produced from 94 mother trees from a single natural forest with up to 5 siblings each, and test population consisted of 71 randomly selected trees. It is therefore very likely that the bias of the shared mother trees affected the accuracy of the predictions. More accurate predictions would probably be obtained if siblings from the same mother trees were distributed evenly between the test and training populations.

### 4.3 Performance of deep learning and gradient boosting decision trees for genomic prediction

CNN1D achieved maximum predictive abilities of 0.082 for D7 and 0.218 for H7. It also achieved the highest predictive ability of any method when using the 96 and 192 SNP datasets for H7 and the lowest predictive ability of any method when using the 48, 96, and 192 SNP datasets for D7 (**Table 2**). Similar results were obtained in a GS study on strawberries and blueberries, in which CNN exhibited better predictive ability than linear models for traits with relatively strong epistatic effects (Zingaretti et al., 2020). In addition, the predictive ability achieved for H7 with RKHS was highest for the 48 SNP dataset and declined as the number of SNPs increased. This implies that the epistatic effects in question are particularly strong among the small number of genes close to the selected 48 SNPs. The predictive accuracy achieved for H7 with CNN1D was highest when using the 96 and 192 SNP datasets, indicating that more genes in the vicinity of these SNPs are involved in regulating this trait. Maximum predictive abilities of 0.108 for D7 and 0.199 for H7 were achieved with CNN2D, in which the haplotype patterns were modeled using the CNN filter (**Table 2**). Although the basic structure of the CNN2D model is similar to that of the CNN1D model, the inclusion of nuclear phase information estimated by beagle seems to weaken the trend observed with the latter model due to the greater complexity of CNN2D. LGB achieved a maximum predictive ability of 0.167 for D7 and 0.162 for H7. It also had the highest predictive ability of any method for D7 when using the 48 and 96 SNP datasets (**Table 2**). An earlier GS study on maize suggested that LGB provides better accuracy than other GBDTs and rrBLUP, and that genome-wide epistatic interactions can be cumulatively learned by LGB given a sufficiently large population (Yan et al., 2021). However, the number of samples in this work (281) was much smaller than in the maize study and may have been insufficient for LGB to cumulatively learn the genome-wide epistasis interactions. The high accuracy achieved for D7 with the 96 and 192 SNP datasets may also be partly due to the bimodal distribution of the phenotypic values for this trait (**Figure 4**). Most models considered in this work assume that the values of the objective variable are normally distributed, and their accuracy may suffer if this assumption is violated. However, LGB models can predict non-unimodal traits without issue. XGB achieved a maximum predictive ability of 0.120 for D7 and 0.191 for H7; both of these values are lower than those achieved with linear models in most cases (**Table 2**). However, in genomic predictions using simulated data, the predictive ability of XGB exceeded those of BayesB, GBLUP, RF, CNN, and MLP when dominant and epistasis effects were present in addition to additive genetic effects (Abdollahi-Arpanahi et al., 2020). However, XGB achieved poor predictive ability when applied to real data in this work even though the results obtained with RKHS and RF suggest that the selected SNPs (for H7) and all SNPs (for both D7 and H7) have nonadditive genetic structures. Given the very large sample size of the simulation study (over 10,000 samples), this discrepancy may be due to the low number of samples included in this work.

### 4.4 Influence of marker selection on genomic prediction and detection of genetic effects

Predictive ability varied with the number of included SNPs in the order 18037 (all) > 192 > 96 > 48 SNP for D7 and 48 > 96 > 192 > 18037 (all) for H7, and its difference were statistically significant in a few modeling methods (**Table 2**). The Q-Q plots of $-{\text{log}}_{10}\left(P\right)$ values suggest that false positives did not unduly affect the GWAS results for any training population (**Figure S2**). For H7, the higher predictive ability of the models with SNP selection compared to those using the full SNP dataset may be due to the elimination of noise introduced by unlinked SNPs. ddRAD-seq randomly selects DNA fragments from the whole genome with a certain density and therefore includes some SNPs that are not linked to the target trait. The $-{\text{log}}_{10}\left(P\right)$ values of such SNPs should be very small in GWAS, and removing such SNPs should increase model accuracy. The improvement in prediction accuracy due to the decrease in the number of markers was particularly noticeable in H7, and tree height might be relatively less complex than tree diameter D7. Although the complexity of gene regulation in primary and secondary growth should vary depending on tree species, secondary growth is likely to involve a large number of genes compared to primary growth due to the greater diversity of cell types and processes associated with the secondary growth in general (Oh et al., 2003; Demura and Fukuda, 2007). The difference of complexity of gene regulation might be main reason of the opposite tendencies between H7 and D7.

Other genomic prediction studies on disease resistance in aquaculture species have similarly found that SNP marker selection improves the accuracy of genomic prediction models (Luo et al., 2021). The predictive ability for D7 exceeded that for H7 irrespective of the chosen method when using the full SNP dataset (**Table 2**), possibly because the genetic heritability of D7 exceeds that of H7. However, the highest accuracy for H7 was achieved when using 48 selected SNPs whereas the highest accuracy for D7 was achieved with the full set of SNPs (**Table 2**). This may be related to the fact that the phenotypic values for H7 were normally distributed whereas those for D7 were bimodal (**Figure 4**). Because the first stage in the SNP analysis was performed using a linear model with FASTmrEMMA, it is possible that the pipeline was best suited for pseudo-normally distributed focal variables like H7 and was thus more effective at detecting significant SNPs for H7 than for D7.

The differences in predictive accuracy between semiparametric and nonparametric methods were greater than those between parametric methods (**Table 2**). Parametric methods only consider additive genetic effects, whereas nonparametric methods do not exclude other genetic effects such as dominance and epistasis and thus produce models that can account for a wider range of genetic effects. This is consistent with the findings of Howard et al. (2014), whose studies using simulated data showed that four semiparametric/nonparametric methods achieved markedly differing accuracies that were related to their ability to explain both additive and epistatic genetic structure. The differences in predictive ability between the tested methods thus suggest that the SNPs selected for H7 have a relatively strong epistatic genetic structure but those selected for D7 may have a more additive genetic structure. These results show that to maximize predictive ability when constructing a genomic prediction model based on GWAS, it is important to select a method appropriate for the genetic structure of the selected SNPs.

## 5 Conclusion

Genomic prediction models were constructed for two growth traits in the tropical timber tree species *S. macrophylla*: DBH (D7) and HT (H7) in the seventh year from transplantation at the PT. Sari Bumi Kusuma forest concession in central Kalimantan, Indonesia. Unlike species with established lineages and pedigrees such as crops and livestock, the studied progeny trial retains high genetic diversity due to the large effective population size of mother trees and the reliance on open-pollinated mating to regenerate the reproductive source of the trial. Despite this high genetic diversity, the measured values correlated positively with the outputs of genomic prediction models for D7 and H7. When GWAS data were used to select SNP subsets, different sets of SNPs were selected for each split of the training/test population and the highest predictive accuracies were achieved with the full set of SNPs for D7 and 48 selected SNPs for H7, these opposite tendencies might be originated from difference of genetic architecture between primary and secondary growth of the species. These results show that the SNP subset with the highest predictive ability in H7 can be used for genotyping next-generation populations in breeding programs in order to reduce costs while maximizing genetic gains. Although it is necessary to further increase the marker density and the power of LD detection by increasing the number of individuals, the genomic prediction models and subsequent selection at seedling stage using the models show potential to accelerate breeding cycles for non-cultivated tree species.

## Data availability statement

The datasets presented in this study can be found in online repositories. The names of the repository/repositories and accession number(s) can be found below: https://www.ncbi.nlm.nih.gov/bioproject/PRJNA988453/; https://doi.org/10.5281/zenodo.8051012; https://doi.org/10.5061/dryad.kkwh70s8d.

## Author contributions

NT, KU, and YT conceived the ideas of the project. MN and SP established progeny trials and scientific data from the progeny trials were obtained by MN, SP, W, and SI, and NT, MN, W, SI, S, and SP carried out field work. NT, MN, W, SI, S, and YT contributed to searching for funds. HA, NT and KU performed the experiment and data analysis and HA and NT wrote the first draft of the manuscript and contributed to the writing and revision of the manuscript. All authors have approved the manuscript for publication. All authors contributed to the article.
