# Supplementary material for: Comparing modeling methods of genomic prediction for growth traits of a tropical timber species, Shorea macrophylla
Source: Front Plant Sci. 2023 Oct 31;14:1241908. doi: 10.3389/fpls.2023.1241908 (PMC10644202; doi:10.3389/fpls.2023.1241908)
Supplement: Supplementary file 1 [file Image_1.pdf]

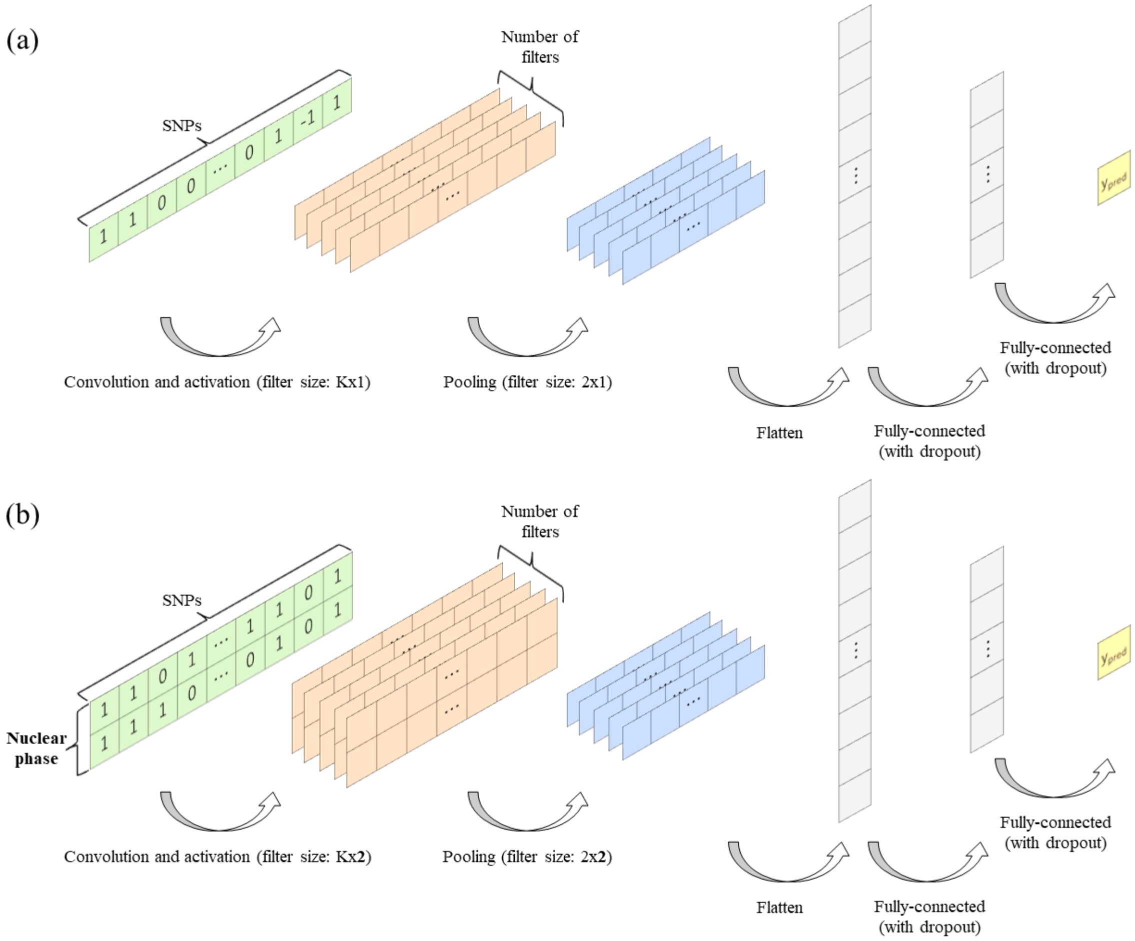

Figure S1. Simplified architectures of CNN1D and CNN2D within a sample.

Simplified architectures of (a) CNN1D and (b) CNN2D. Genotype values in CNN1D were scaled in actual analyses.
