# Supplementary material for: Comparing modeling methods of genomic prediction for growth traits of a tropical timber species, Shorea macrophylla
Source: Front Plant Sci. 2023 Oct 31;14:1241908. doi: 10.3389/fpls.2023.1241908 (PMC10644202; doi:10.3389/fpls.2023.1241908)
Supplement: Supplementary file 2 [file Image_2.pdf]

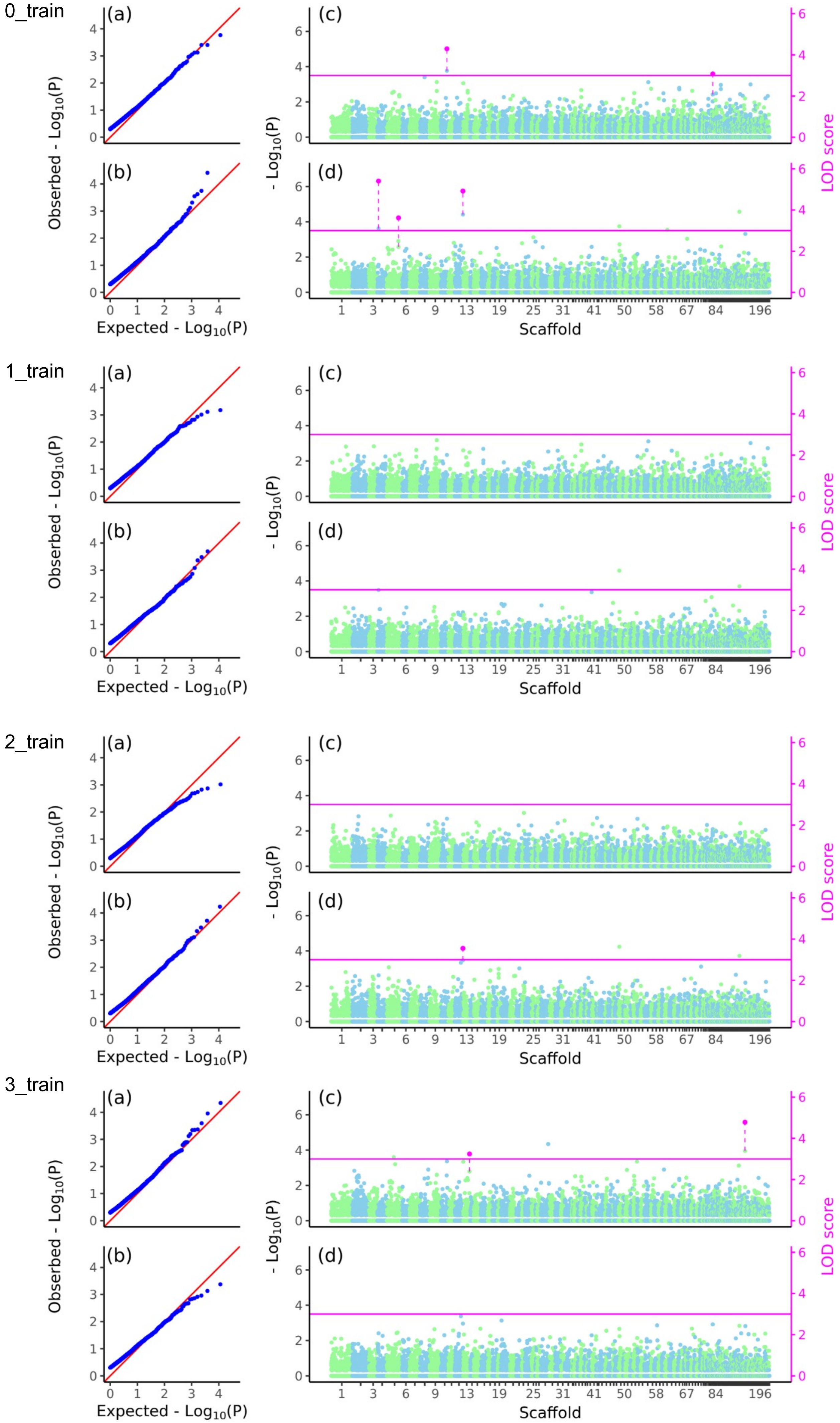
0_train
1_train
2_train
3_train
(a)
(b)
(c)
(d)
Obserbed - Log10(P)
Expected - Log10(P)
- Log10(P)
LOD score
Scaffold
1 3 6 9 13 19 25 31 41 50 58 67 84 196

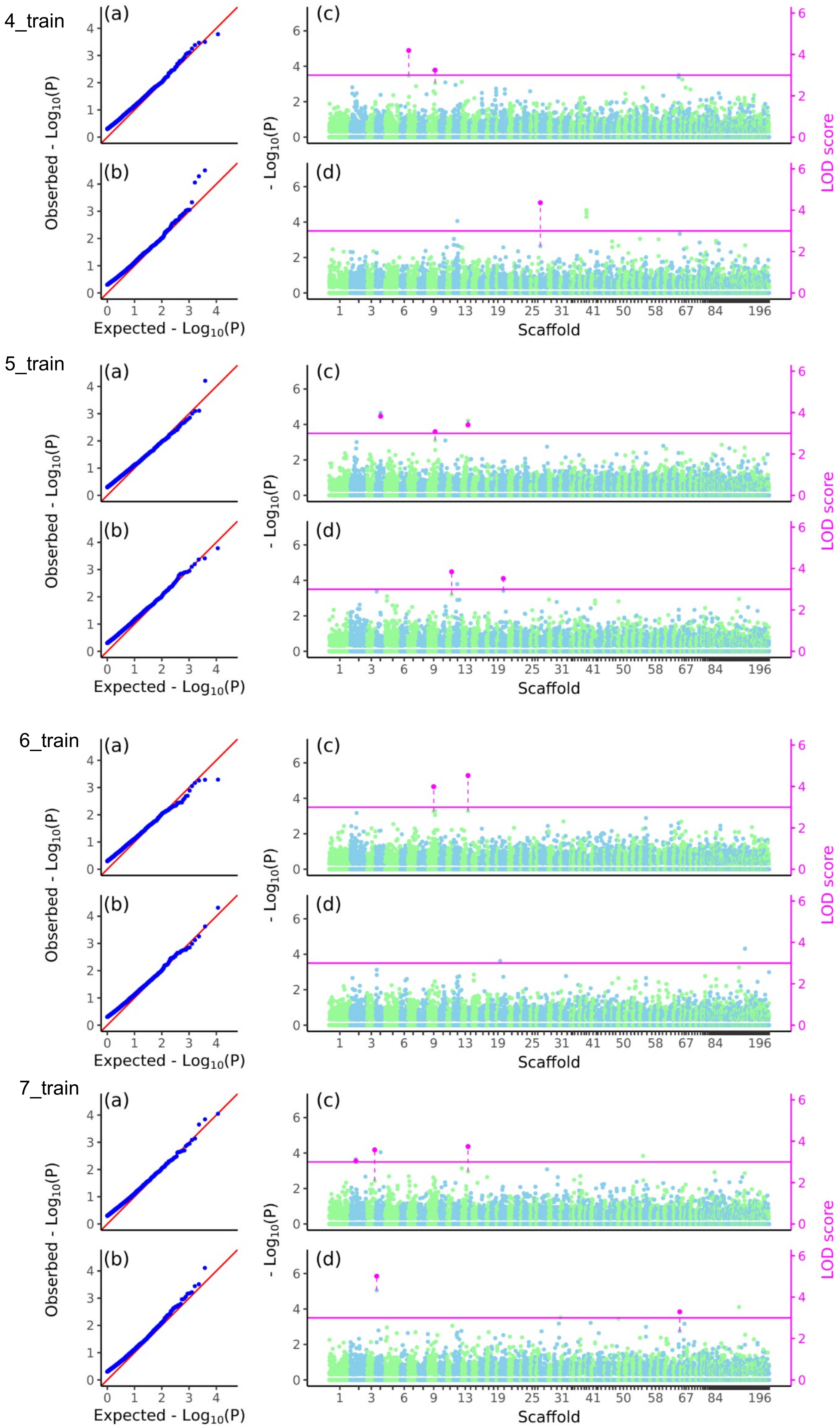
4_train
5_train
6_train
7_train
(a)
(b)
(c)
(d)
Obserbed - Log_{10}(P)
Expected - Log_{10}(P)
- Log_{10}(P)
LOD score
Scaffold
1
3
6
9
13
19
25
31
41
50
58
67
84
196

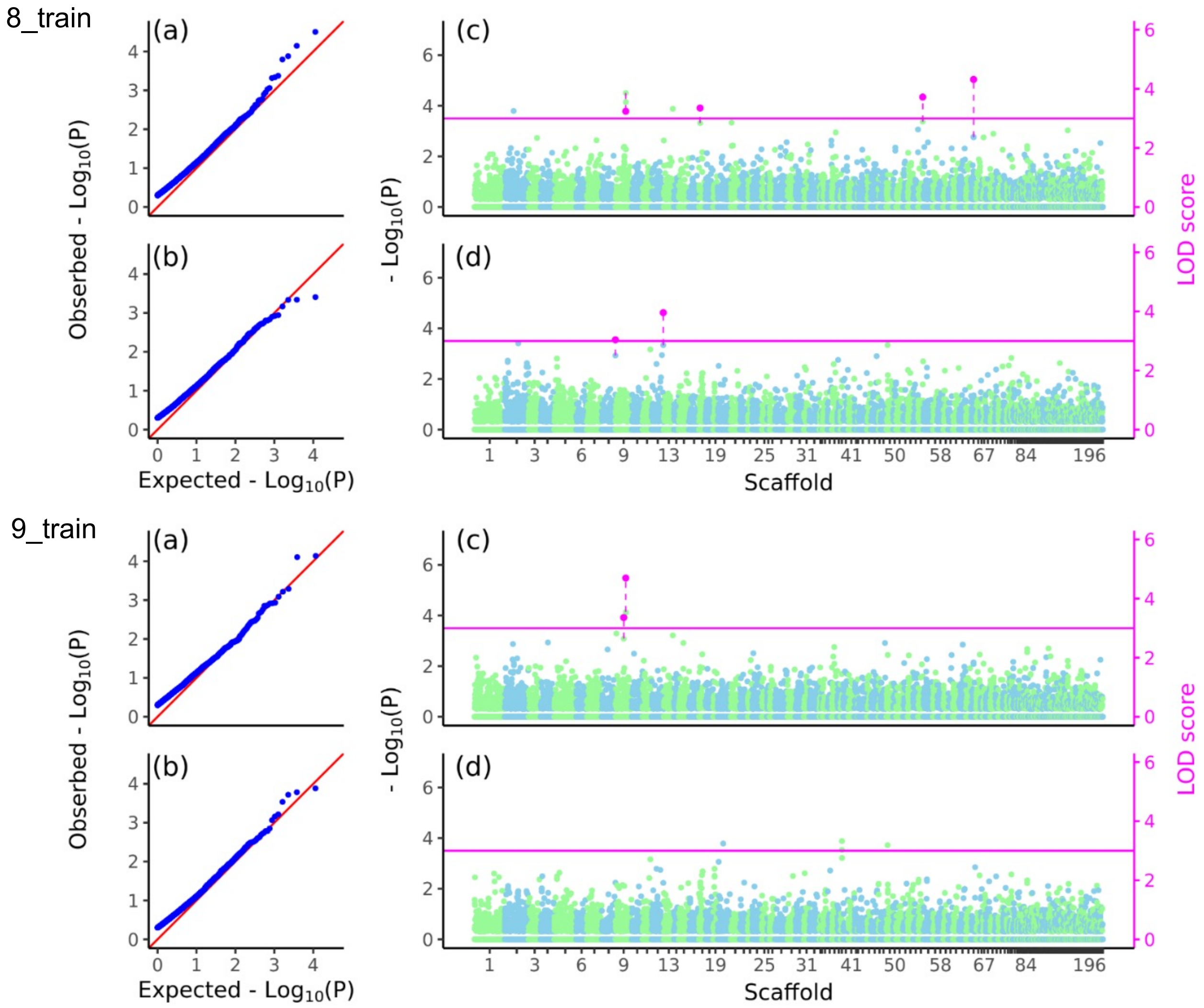

Figure S2. Q-Q and Manhattan plots of the second GWAS using SNPs, D7 and H7.

(a) and (b) represent Q-Q plots for D7 and H7, respectively and (c) and (d) represent Manhattan plots for D7 and H7, respectively.
