# Supplementary material for: Comparing modeling methods of genomic prediction for growth traits of a tropical timber species, Shorea macrophylla
Source: Front Plant Sci. 2023 Oct 31;14:1241908. doi: 10.3389/fpls.2023.1241908 (PMC10644202; doi:10.3389/fpls.2023.1241908)
Supplement: Supplementary file 3 [file Table_1.docx]

Table S1. Parameters for optimization in XGB and LGB.

| Parameter | XGBoost | LightGBM | Description |
| --- | --- | --- | --- |
| max_depth | int 6 – 11 | - | Maximum depth of a tree. |
| Num_leaves | - | int 2 – 60 | Maximum number of leaves in a tree. |
| Learning_rate | float 0 – 1 | float 1e-6 – 1 | Step size shrinkage used in update. |
| Gamma | float 0 – 1 | - | Minimum loss reduction required to make a further partition on a leaf node of the tree. |
| Min_child_weight | float 0 – 10 | float 1e-6 – 1 | Minimum sum of instance weight needed in a child. |
| Subsample | float 0 – 1 | float 0.4 – 1 | Subsample ratio of the training instances. |
| Subsample_freq | - | int 1 – 7 | Subsample frequency in iterations. |
| Colsample_bytree | float 0 – 1 | float 0.4 – 1 | Subsample ratio of columns. |
| Reg_alpha | float 0 – 1 | float 1e-6 – 1 | L1 regularization term on weights. |
| Reg_lambda | float 0 – 1 | float 1e-6 – 1 | L2 regularization term on weights. |

“int” and “float” denote integer and floating-point values. Parameter names are taken from the scikit-learn API of each library.

“learning_rate”, “min_child_weight”, “reg_alpha” and “reg_lambda” in LGB were sampled from a logarithmic scale.
