# Supplementary material for: Comparing modeling methods of genomic prediction for growth traits of a tropical timber species, Shorea macrophylla
Source: Front Plant Sci. 2023 Oct 31;14:1241908. doi: 10.3389/fpls.2023.1241908 (PMC10644202; doi:10.3389/fpls.2023.1241908)
Supplement: Supplementary file 4 [file Table_2.docx]

Table S2. Parameters for optimization in CNN1D and CNN2D

| Parameter | CNN1D, CNN2D | Description |
| --- | --- | --- |
| filters | int 16 – 256 | The number of filters in convolutional layers |
| kernel_size | int 3 – 9 | Kernel size of convolutional layers |
| dropout0 | float 0 – 0.5 | Dropout rate before a Fully connected layer |
| dropout1 | float 0 – 0.5 | Dropout rate before a Fully connected layer |
| dropout2 | float 0 – 0.5 | Dropout rate before a Fully connected layer |
| dense | int 16 – 256 | The number of units in Fully connected layers |
| lr | float 1e-4 –1e-3 | Learning rate for the optimizer |

“lr” was sampled from a logarithmic scale.
