# Supplementary material for: Comparing modeling methods of genomic prediction for growth traits of a tropical timber species, Shorea macrophylla
Source: Front Plant Sci. 2023 Oct 31;14:1241908. doi: 10.3389/fpls.2023.1241908 (PMC10644202; doi:10.3389/fpls.2023.1241908)
Supplement: Supplementary file 5 [file Table_3.docx]

Table S3. Architectures for CNN1D and CNN2D

| CNN1D | | CNN2D | |
| --- | --- | --- | --- |
| Type of layer | Output shape | Type of layer | Output shape |
| Input | N, P, 1 | Input | N, P, 2, 1 |
| Conv1D | N, P, F | Conv2D | N, P, 2, F |
| Conv1D | N, P, F | Conv2D | N, P, 2, F |
| MaxPooling1D | N, P / 2, F | MaxPooling2D | N, P / 2, 1, F |
| Conv1D | N, P / 2, 2 * F | Conv2D | N, P / 2, 1, 2 * F |
| Conv1D | N, P / 2, 2 * F | Conv2D | N, P / 2, 1, 2 * F |
| MaxPooling1D | N, P / 4, 2 * F | MaxPooling2D | N, P / 4, 1, 2 * F |
| Flatten | N, P * F / 2 | Flatten | N, P * F / 2 |
| Dropout | N, P * F / 2 | Dropout | N, P * F / 2 |
| Dense | N, 2 * D | Dense | N, 2 * D |
| Dropout | N, 2 * D | Dropout | N, 2 * D |
| Dense | N, D | Dense | N, D |
| Dropout | N, D | Dropout | N, D |
| Dense | N, 1 | Dense | N, 1 |

Layer types and the corresponding output shapes are shown on each row. Names of layer types are taken from the Keras library. “N”, “P”, “F”, and “D” denote batch size, number of SNPs, number of filters on the convolution layer, and number of units on the fully-connected layer, where F and D were optimized using the Optuna library.
